# A Comparison of Free-Standing versus Co-Located Long-Term Acute Care Hospitals

**DOI:** 10.1371/journal.pone.0139742

**Published:** 2015-10-06

**Authors:** Jeremy M. Kahn, Amber E. Barnato, Judith R. Lave, Francis Pike, Lisa A. Weissfeld, Tri Q. Le, Derek C. Angus

**Affiliations:** 1 Clinical Research, Investigation and Systems Modeling of Acute Illness (CRISMA) Center, Department of Critical Care Medicine, University of Pittsburgh School of Medicine, Pittsburgh, Pennsylvania, United States of America; 2 Department of Health Policy & Management, University of Pittsburgh Graduate School of Public Health, Pittsburgh, Pennsylvania, United States of America; 3 Center for Research on Health Care, Division of General Internal Medicine, University of Pittsburgh School of Medicine, Pittsburgh Pennsylvania, United States of America; 4 Department of Biostatistics, University of Pittsburgh Graduate School of Public Health, Pittsburgh Pennsylvania, United States of America; 5 Statistics Collaborative, Inc. Washington, D.C., United States of America; Oregon Health and Science University, UNITED STATES

## Abstract

**Background:**

Long-term acute care hospitals (LTACs) provide specialized treatment for patients with chronic critical illness. Increasingly LTACs are co-located within traditional short-stay hospitals rather than operated as free-standing facilities, which may affect LTAC utilization patterns and outcomes.

**Methods:**

We compared free-standing and co-located LTACs using 2005 data from the United States Centers for Medicare & Medicaid Services. We used bivariate analyses to examine patient characteristics and timing of LTAC transfer, and used propensity matching and multivariable regression to examine mortality, readmissions, and costs after transfer.

**Results:**

Of 379 LTACs in our sample, 192 (50.7%) were free-standing and 187 (49.3%) were co-located in a short-stay hospital. Co-located LTACs were smaller (median bed size: 34 vs. 66, p <0.001) and more likely to be for-profit (72.2% v. 68.8%, p = 0.001) than freestanding LTACs. Co-located LTACs admitted patients later in their hospital course (average time prior to transfer: 15.5 days vs. 14.0 days) and were more likely to admit patients for ventilator weaning (15.9% vs. 12.4%). In the multivariate propensity-matched analysis, patients in co-located LTACs experienced higher 180-day mortality (adjusted relative risk: 1.05, 95% CI: 1.00–1.11, p = 0.04) but lower readmission rates (adjusted relative risk: 0.86, 95% CI: 0.75–0.98, p = 0.02). Costs were similar between the two hospital types (mean difference in costs within 180 days of transfer: -$3,580, 95% CI: -$8,720 –$1,550, p = 0.17).

**Conclusions:**

Compared to patients in free-standing LTACs, patients in co-located LTACs experience slightly higher mortality but lower readmission rates, with no change in overall resource use as measured by 180 day costs.

## Background

Long-term acute care hospitals (LTACs) are a unique type of inpatient health care facility in the United States (US), providing specialized care to acutely ill patients requiring extended hospitalizations [1]. Defined by the US Centers for Medicare and Medicaid Services (CMS) as acute care hospitals with an average length of stay greater or equal to 25 days, LTACs were formally established in the early 1980’s during the shift towards prospective payment for US hospitals, under which hospitals are paid a set amount for each payment rather than have payments determined by costs [2]. At the time, the creators of the prospective payment system realized that a small subset of hospitals, typically respiratory hospitals with a focus on inpatient rehabilitation of patients with chronic respiratory needs, were systematically different than other acute care hospitals and might not be financially viable were they to be paid under the same prospective payment system. CMS therefore exempted hospitals with long geometric mean lengths of stay from prospective payment, codifying into payment policy the class of hospitals known as LTACs.

Over time, LTACs have evolved from purely respiratory-focused hospitals to hospitals that provide care for patients with all types of chronic critical illness [3]. Generally, this means that patients are transferred from short stay hospitals to LTACs when they have recovered from their acute illness (and therefore don’t require all the resources of an acute care hospital) but still have substantial inpatient needs (and therefore can’t be transferred to other types of post-acute care such as skilled nursing facilities or inpatient rehabilitation hospitals) [4]. Most commonly, patients are transferred for prolonged mechanical ventilation necessitating intensive ventilator weaning, severe wounds, the need for ongoing hemodialysis and other low-intensity organ support, and other types of chronic critical illness, directly from traditional short stay hospitals [5]. Although LTACs are unique to the United States, many industrial nations have similar types of hospitals that focus on the care of the long-term ventilator patients [6,7].

LTACs are among the fastest growing segments of the health care system [8]. The number of LTACs increased from 277 in 2003 to 412 in 2010, with Medicare spending on LTACs during this period increasing from $2.7 billion to $5.2 billion [9]. The reasons for this increase are complex but may be attributable to several primary factors. First, the prevalence of prolonged mechanical ventilation is increasing due to the aging of the population and advances in the care of critically ill patients that leads to greater survivorship but also greater short-term disability [10]. Second, the US prospective payment system creates financial incentives for LTAC transfer, since hospitals can increase revenue by discharging patients “sicker and quicker” but still receiving the same payment [11]. As a consequence of this growth, LTACs are an important policy problem in the United States.

Historically most LTACs operated as free-standing health care facilities. However, most of the recent growth in LTACs has occurred in the form of co-located hospitals, also known as “hospitals within a hospital” [9]. Co-located LTACs differ from free-standing LTACs in that they are physically located within traditional short stay hospitals even though they are organizationally, managerially and financially independent from their host hospital. This arrangement may increase efficiency by allowing them to share some resources with their host hospital such as radiological and procedural services. However, co-location also reduces the barriers to LTAC transfer, potentially leading to unnecessary transfers and overutilization.[12] Concerned about potential overutilization, Medicare has advanced plans to limit transfers to co-located LTACs, reducing payments if more than 25% of co-located LTACs patients come from a single hospital [9].

This policy, known as the “25% rule” has not yet been fully implemented due to repeated Congressional delays—current regulations reduce payments if transfer thresholds exceed 50% for co-located LTACs. Moreover current proposals for the 25% rule include all LTACs, not just free-standing LTACs. Yet if fully implemented, this policy will ultimately discourage the use of co-located LTACs. Yet it is not clear whether the co-located LTAC model should be encouraged or discouraged, since the actual differences between co-located and free-standing LTACs are not well described. Co-location, a form of vertical integration, could improve outcomes and lower costs by allowing LTACs to afford themselves of the specialized services of short stay hospitals [13]. Co-location could also worsen outcomes and increase costs since co-located LTACs are typically smaller than free-standing LTACs and thus have less clinical experience in the care of critically ill patients [14]. To better understand these issues, we used data on fee-for-service Medicare beneficiaries to compare patient characteristics and outcomes between free-standing and co-located LTACs.

## Methods

### Study design and data

We performed a retrospective cohort study of Medicare beneficiaries transferred from United States short stay hospitals to LTACs in calendar year 2005. We obtained patient-level data from the Medicare Provider Analysis and Review (MedPAR) file, which contains the final action hospitalization claims for all fee-for-service Medicare beneficiaries in the United States, including both short stay hospitals, skilled nursing facility (SNF) admissions, and LTACs. Medicare is the payer for over two-thirds of all LTAC hospitalizations and is the only national source of data for LTAC admissions [1]. We obtained hospital-level data from the CMS Healthcare Cost Report Information System (HCRIS). Short stay hospitals and LTACs were identified in HCRIS, as previously described [15].

### Patients

All patients admitted to a short stay hospital during 2005 were initially eligible. To create a homogenous cohort we limited the analysis to patients aged 66 and over and patients who were community dwelling prior to their short stay hospital admission. We defined community dwelling as not being admitted to a short-stay hospital, SNF or LTAC immediately prior to their index short stay hospital admission, i.e. nursing home residents and patients at home were considered community dwelling. To avoid interdependence of observations we analyzed only the first short-stay hospital admission for each patient.

### Variables

The primary exposure variable was the type of LTAC to which the patient was transferred: either free-standing or co-located. We identified LTAC transfers by directly observing them in the claims rather than using the “discharge location” field in MedPAR which is known to be inaccurate [15]. We identified co-located LTACs in two ways. First, we used a preliminary listing of co-located LTACs provided by the Centers for Medicare & Medicaid Services. Second, we geocoded all US short stay hospitals and LTACs using ArcGIS software (ESRI, Redlands, California) based on their street address listed in HCRIS. We then calculated the linear arc distance between each LTAC and the nearest short-stay hospital. For LTACs located less than one mile to the nearest short-stay hospital we performed internet searches to determine the LTAC type. We reconciled differences between these two lists by making direct phone calls to the hospitals.

The primary outcome variables were mortality (30, 90 and 180 days, measured from the date of transfer to the LTAC); all-cause hospital readmissions (which we defined in two ways: within 30-days of LTAC transfer whether or not the patient remained at the LTAC; and during the LTAC stay regardless of timing); LTAC length of stay in days; LTAC costs, and 180-day hospitalization-related costs from the date of LTAC transfer, including LTAC costs and additional short stay hospital or skilled nursing costs. These variables were chosen because of their importance to patients, in the case of mortality; and society, in the case of readmissions, length of stay and costs. Mortality was calculated from observed death dates in the Medicare Beneficiary Summary Files. Readmissions and LTAC length of stay were determined directly from the claims. Costs were determined from total charges reported in the claims multiplied by hospital-specific cost-to-charge ratios from HCRIS [16]. Because we were interested in readmissions and length of stay due to their relation to resource use, we did not specifically address the potential for informative censoring due to mortality. Instead, these outcomes are meant to be considered separately.

### Analysis

We designed the analysis to answer three questions: (1) do LTAC types differ in organizational characteristics? (2) do LTAC types differ in the types of patients admitted and the timing of transfer for admitted patients? and (3) do patient outcomes differ between LTAC types? To answer the first question we compared LTAC characteristics between free-standing and co-located LTACs using two-sample t-tests, chi-square tests and Wilcoxon rank-sum tests, as appropriate. To answer the second question we compared patient characteristics by transfer destination, either free-standing or co-located LTAC. For this analysis we did not perform statistical hypothesis testing, since due to the large number of patients any differences would be statistically significant even if not clinically significant.

To answer the third question, we compared patient outcomes between free-standing and co-located LTACs. To minimize confounding, for this analysis we limited the cohort to patients with the 10 most common diagnoses resulting in an LTAC transfer, using diagnosis related group (DRG) from the short stay hospital. These 10 diagnoses accounted for 42.5% of all LTAC transfers during this year. We further limited the analysis to Dartmouth Atlas Hospital Referral Regions with both types of LTACs; short stay hospitals that transferred patients to both types of LTACs; and short stay hospitals with at least 25 eligible patients.

Patients admitted to a short-stay hospital containing a co-located LTAC are both more likely to be transferred to an LTAC in general and more likely to be transferred to a co-located LTAC in particular [12], and both of these decisions may be associated with patient outcomes. We used a propensity score to account for the bias inherent in this comparison [17,18]. First, we created a propensity score for transfer to a co-located LTAC using a multivariable regression model that included all patient and hospital factors potentially related to LTAC transfer. We derived the score based on all eligible patients, including those not-transferred to an LTAC, so that the propensity score would best estimate the probability of admission to an LTAC type; fitting a multinomial regression model in which the outcome was the entire range of potential outcomes: dead or hospice, home, skilled nursing facility, another short-stay hospital, a co-located LTAC, or a free standing LTAC. Thus, the propensity score accounted for not only the propensity to be admitted to a co-located LTAC but also the propensity to be transferred to an LTAC in general, instead of being transferred to a post-acute care facility or dying in the hospital.

After creating the propensity score we excluded patients who were not transferred to an LTAC. We then matched the remaining patients based on their propensity to be transferred to a co-located LTAC, using a combined nearest-neighbor and Mahalanobis matching procedure [19]. As a result, the analytic cohort in this step contained only patients who were transferred to an LTAC and were of equal likelihood to be transferred to a co-located LTAC. In this cohort we then performed multivariable regression examining the relationship between LTAC type and outcomes, controlling for patient and short-stay hospital characteristics. We used generalized linear models to account for the different distributional forms of the outcome variables. Additional details regarding the propensity matching and regression analysis, including an expanded rationale for this approach, are available in the S1 File.

Analyses were performed with SAS 9.3 (SAS Institute, Cary, NC). All statistical tests were two-tailed, and a p-value of ≤0.05 was considered significant. This work used existing de-identified data and was considered exempt from the requirement for informed consent by the University of Pittsburgh Institutional Review Board. All data related to this manuscript are publically available from the United States Centers for Medicare and Medicaid Services, subject to an application and data use agreement (www.resdac.org).

## Results

The study included 379 LTACs of which (49.3%) were co-located in a short stay hospital. Compared to free-standing LTACs, co-located LTACs were smaller; were more likely to be for-profit; and, on average, admitted patients from fewer short-stay hospitals (**Table 1**).

**Table 1:** Long-term acute care hospital characteristics by type.

Characteristic	Free-standing (n = 192)	Co-located (n = 187)	P-value
Bed size	66 [40–109]	34 [30–44]	<0.0001
Ownership			0.001
For-profit	132 (68.8)	135 (72.2)	
Non-profit	40 (20.8)	49 (26.2)	
Government	20 (10.4)	3 (1.6)	
Region			0.0002
Northeast	32 (16.7)	18 (9.4)	
South	95 (49.5)	114 (59.4)	
Midwest	35 (18.2)	47 (24.5)	
West	30 (15.6)	8 (4.2)	
Number of different short stay hospitals from which patients were admitted	18.4 ± 8.7	12.8 ± 6.6	<0.001
Total admissions in MedPAR*	228 [95–361]	182 [114–251]	0.008

Values are median [interquartile range], frequency (percent) or mean ± standard deviation.

MedPAR = Medicare Provider Analysis and Review File.

*The total number of 2005 admissions in MedPAR independent of eligibility for this study.

The analysis of patient differences between hospital types included 77,170 patients in these 379 LTACs. Compared to patients admitted to free-standing LTACs, patients admitted to co-located LTACs were more likely to have been in an ICU in the short stay hospital, were more likely to be transferred for ventilator weaning, and had longer hospital stays (**Table 2**).

**Table 2:** Patient characteristics for all patients transferred to long-term acute care hospitals.

	Free-standing (n = 44,973)	Co-located (n = 32,197)
Age	78.5 ± 7.4	77.8 ± 7.2
Female (%)	25554 (56.8)	17798 (55.3)
Race		
White	34376 (76.4)	25272 (78.5)
Black	7246 (16.1)	5464 (17.0)
Other	3351 (7.5)	1461 (4.5)
Admitted to ICU at short stay hospital	19001 (42.2)	16257 (50.5)
MV status		
Short stay only	4446 (9.9)	3778 (11.7)
Short stay and LTAC	5557 (12.4)	5106 (15.9)
LTAC only	847 (1.9)	595 (1.8)
Neither	34123 (75.9)	22718 (70.6)
Length of Stay		
ICU	7.6 ± 11.7	8.9 ± 12.0
Hospital	14.0 ± 12.6	15.5 ± 12.4
Diagnosis related group		
Tracheostomy with MV 96 (541/542)	5931 (13.2)	5396 (16.8)
Septicemia (416)	2357 (5.2)	1640 (5.1)
Intracranial hemorrhage (14)	2016 (4.5)	910 (2.8)
Pneumonia with CC (89)	1894 (4.2)	981 (3.0)
Respiratory diagnosis with MV (475)	1554 (3.5)	1237 (3.8)
Heart failure and shock (127)	1736 (3.9)	1048 (3.3)
COPD (88)	1472 (3.3)	642 (2.0)
Major bowel procedure with CC (148)	993 (2.2)	1098 (3.4)
Respiratory infection/inflammation w/ CC (79)	1218 (2.7)	840 (2.6)
OR procedure for infection (415)	972 (2.2)	1000 (3.1)
All others	24830 (55.1)	17405 (54.1)

ICU = intensive care unit; LTAC = long-term acute care hospital; MV = mechanical ventilation; CC = comorbidity or complication; COPD = chronic obstructive lung disease

**Fig 1:**
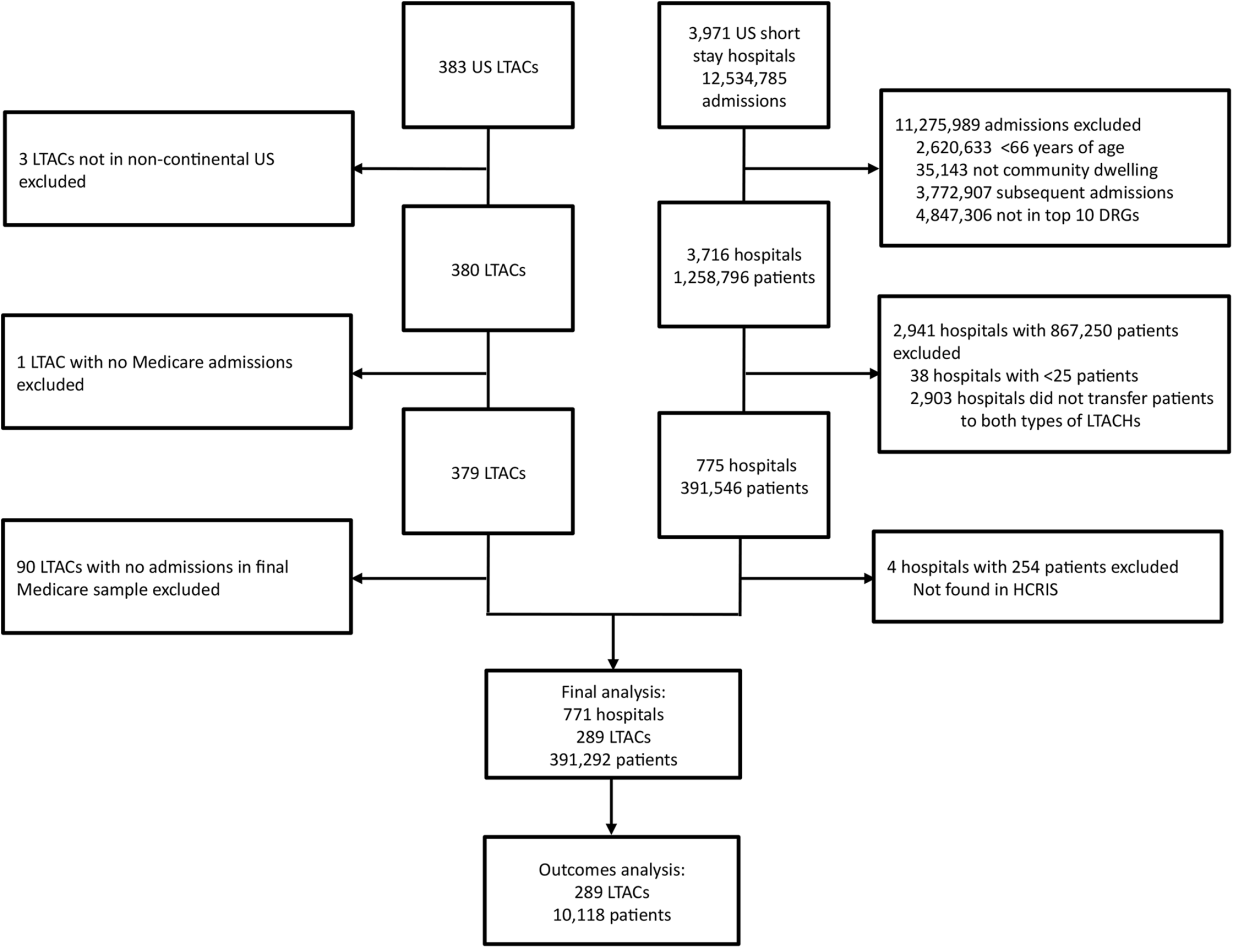
Flow diagram of hospitals and patients. The LTAC-level analysis (Table 1) contains the 379 LTACs in the continental United States with at least 1 Medicare admission. The patient level analyses examining patient characteristics between LTAC types (Table 2) also contains patients in these 379 LTACs. The outcomes analysis (Tables 3 and 4) contains the 289 LTACs in the final analysis and 10,118 patients in the matched sample. LTAC = long-term acute care hospital; DRG = diagnosis related group; HCRIS = Healthcare Cost Reporting Information System.

**Fig 2:**
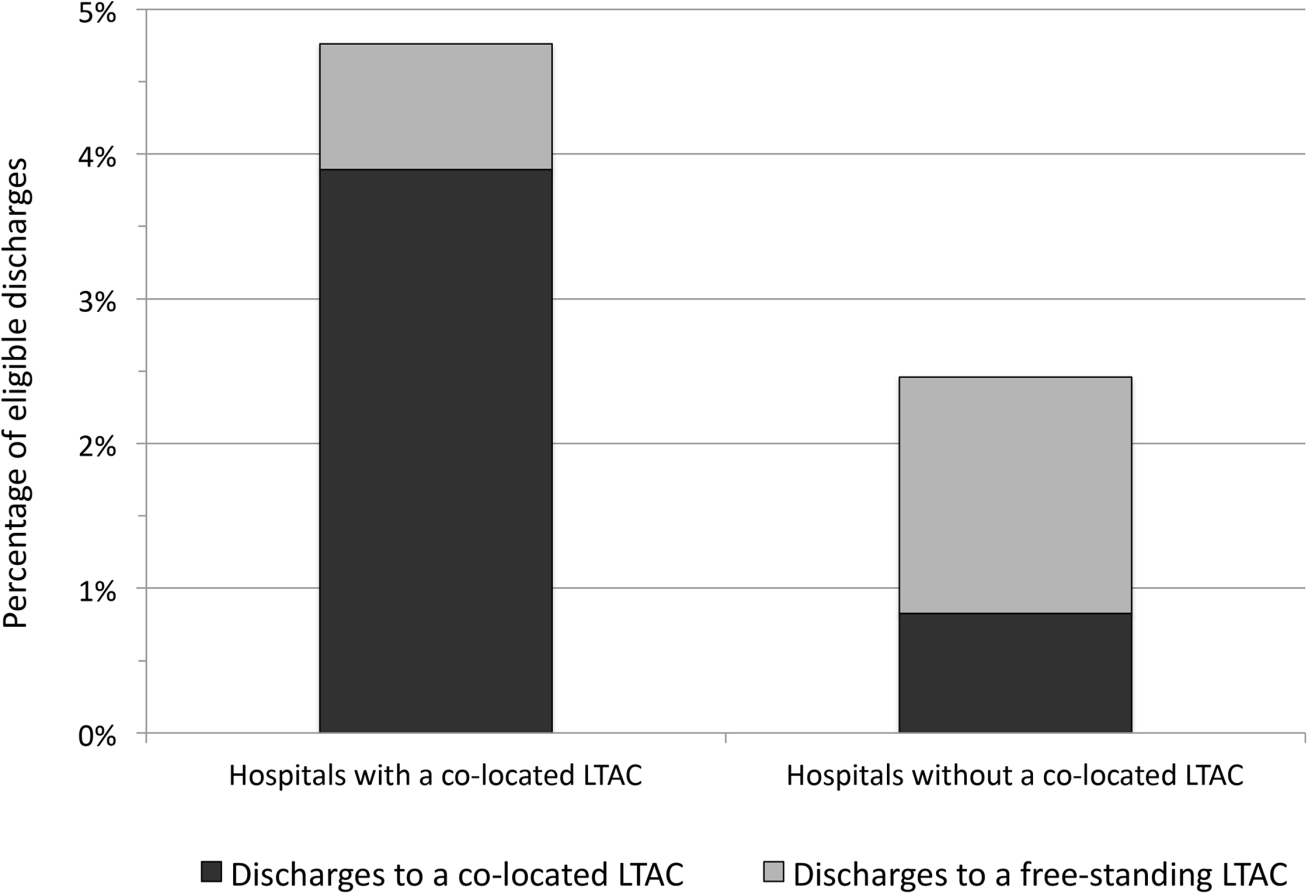
Discharge destination of eligible patients, limited to patients transferred to a long-term acute hospital, by originating hospital type (n = 11,084). LTAC = long-term acute care hospital.

The analysis of outcome differences between LTAC types included 289 LTACs that admitted 391,292 patients from 771 acute care hospitals (Fig 1). Of the 391,292 eligible patients, 322,692 (82.5%) were admitted to short stay hospitals without a co-located LTAC and 68,330 (17.5%) were admitted to short stay hospitals with a co-located LTAC. As in previous studies [12], patients in hospitals with a co-located LTAC were more likely to be transferred to an LTAC than patients in hospitals without a co-located LTAC (4.8% versus 2.5%, p<0.001), and were also more likely be transferred to a co-located LTAC (81.7% versus 33.6% of all LTAC transfers, p<0.001) (Fig 2). Overall, 48.8% of patients admitted to a co-located LTAC were admitted from the host hospital as opposed to a different hospital. An examination of the characteristics and outcomes of all patients with these DRGs, not just those transferred to LTACs, is shown in the S1 File.

Of the 11,084 patients transferred to an LTAC, we excluded 216 patients with the rare transfer pattern of having originated in a hospital containing a co-located LTAC and transferred to a co-located LTAC hosted by a different hospital (i.e. not the LTAC co-located within their hospital). These patients represented 2.0% of all LTAC transfers and 8.2% of all LTAC transfers from hospitals containing a co-located LTAC. This left 10,868 LTAC patients for the propensity matched analysis.

Patient characteristics for the final propensity-matched cohort are shown in **Table 3**. In the matched sample, patients in co-located LTACs were less likely to be mechanically ventilated or have been admitted to an ICU, but overall the cohorts were more similar, compared to patients in free-standing LTACs. Unadjusted and adjusted outcomes in the propensity-matched cohort are shown in **Table 4**and **Table 5**, respectively. Adjusted mortality was higher among patients transferred to co-located LTACs, both at 30, 90 and 180 days. This mortality difference persisted even after controlling for total annual admission volume. Conversely, the risk for readmission was slightly lower, both in 30-days and during the entire LTAC stay. Costs were generally similar between the two groups.

**Table 3:** Patient characteristics for propensity-matched cohort of patients transferred to long-term acute care hospitals. This represents a restricted sample of all patients transferred to LTACs. A table showing all patients eligible for the match, including those not successfully matched, are described in the S1 File.

	Matched Sample	
	Free-standing (n = 5,059)	Co-located (n = 5,059)
Age	78.1 ± 7.4	78.5 ± 7.5
Female (%)	2692 (53.2)	2,829 (55.9)
Race		
White	3,924 (77.6)	3,818 (75.5)
Black	931 (18.4)	969 (19.2)
Other	204 (4.0)	272 (5.3)
Admitted to ICU at short stay hospital	3,455 (68.3)	3,262 (64.5)
Mechanical ventilation status		
Short stay only	994 (19.6)	966 (19.1)
Short stay and LTAC	1,612 (31.9)	1,874 (37.0)
Neither	2,388 (47.2)	2,170 (42.9)
Length of stay in short stay hospital		
ICU	15.2 ± 15.4	14.1 ± 14.9
Hospital	20.3 ± 15.4	18.9 ± 15.0

ICU = intensive care unit; LTAC = long-term acute care hospital

**Table 4:** Unadjusted outcomes for the propensity-matched cohort of patients transferred to long-term acute care hospitals.

	Free-standing (n = 5,059)	Co-located (n = 5,059)
Mortality		
90 days	2,298 (45.2)	2,147 (42.4)
180 days	2,665 (52.7)	2,504 (49.5)
365 days	3,024 (59.8)	2,907 (57.5)
Readmissions, all patients		
30-day	1,643 (32.5%)	1,556 (30.8%)
During LTAC stay	827 (16.4%)	778 (15.4%)
Readmissions, survivors (n = 8,044)		
30-day	1,615 (39.4%)	1,535 (38.9%)
During LTAC stay	766 (18.7%)	724 (18.3%)
Costs		
LTAC stay	$42,959 ± $50,550	$43,613 ± $57,573
180 day	$45,731 ± $64,140	$49,331 ± $66,711

ICU = intensive care unit; LTAC = long-term acute care hospital

**Table 5:** Adjusted outcomes among patients transferred to a co-located LTAC compared to a free-standing LTAC (n = 10,118).*

Outcome measure	Estimate	95% CI	P value
Mortality (RR)			
30 days	1.07	(1.00, 1.15)	0.06
90 days	1.06	(1.00, 1.13)	0.04
180 days	1.05	(1.00, 1.11)	0.04
30-day readmissions (RR)			
All patients	0.91	(0.84, 0.98)	0.02
Survivors only (n = 8,044)	0.94	(0.88, 1.01)	0.09
Readmissions during LTAC stay (RR)			
All patients	0.86	(0.75, 0.98)	0.02
Survivors only (n = 8,044)	0.89	(0.79, 1.01)	0.08
LTAC length of stay, days (mean difference)			
All patients	-1.11	(-2.47, 0.26)	0.11
Survivors only (n = 8,044)	-0.41	(-1.76, 0.94)	0.55
LTAC costs, thousands (mean difference)			
All patients	2.19	(-4.41, 8.79)	0.51
Survivors only (n = 8,044)	3.75	(-2.57, 10.08)	0.24
180 day costs, thousands (mean difference)	-3.58	(-8.72, 1.55)	0.17

*Patients were matched based on their propensity for transfer to a co-located LTAC as well their ventilation status, ICU admission status and the presence of a co-located LTAC in their admission short stay hospital. All models adjust for age, gender, short stay hospitalization length of stay, short stay hospital admission source ventilation status, ICU admission status, patient comorbidities defined in the manner of Elixhauser and annual hospital volume. Models also account for clustering by LTAC using generalized estimating equations.

LTAC = long-term acute care hospital; CI = confidence interval; RR = relative risk

## Discussion

In a national cohort of Medicare beneficiaries we found that admission to a co-located LTAC is associated with increased mortality but decreased hospital readmission rates, even after controlling for patient characteristics and patient selection. We also found that, when considering similar patient types, co-located LTACs tend to admit patients later in their hospital course, and are more likely to admit patients for ventilator weaning, compared to free-standing LTACs.

The higher mortality in co-located LTACs may be due to several factors. Co-located LTACs are smaller than free-standing LTACs, and may not have the economies of scale to avail themselves of intensivist physician staffing [20] or multidisciplinary care teams [21] which are known to save lives in the ICU setting. Additionally, the mortality difference may be due to differences in application of protocols for sedation and weaning from mechanical ventilation [22]. Since co-located LTACs are organizationally distinct from their host hospitals (sharing space and some services but nothing else), they would not be expected to directly obtain this expertise from their host hospitals.

Another possible cause could be the influence of the host hospital itself. If the LTAC clinicians are reassured by the availability of the host hospital’s services and clinicians they may be more reluctant to transfer unstable patients back to the short stay hospital. We also cannot rule out unmeasured differences in severity of illness or other selection–based factors, since our study did not use clinical risk adjustment and the absolute mortality differences were small. We did address whether the observed differences may be due to the difference in volume itself, since volume-outcome relationships are well described in patients with critical illness [14]. Our mortality estimates persisted after adjusting for annual volume, making this explanation unlikely.

The lower readmission rate we observed is potentially due to the fact that co-located LTACs can avail themselves of the services of short stay hospitals in the care of their patients, including subspecialty consults and radiological evaluation. Patients in co-located LTACs requiring these services need not be transferred back to short stay hospitals to receive them, reducing the need for readmission. Under a prospective-payment model, this benefit might reduce LTAC costs to payers, who then do not have to pay for additional hospital admissions. Nonetheless, it does not appear to reduce overall costs, which were not significantly different between the two study types. Alternatively, this finding could be due to competing risks, since we observed increased mortality in co-located hospitals and decedents are no-longer at risk for readmissions.

Co-located LTACs tended to admit more sick patients overall (in that they were more likely to have been in in ICU and more likely to have been mechanically ventilated), and admit patients later in their hospital course, even after minimizing differences between the two groups through propensity matching. This finding highlights the fact that there is little standardization about when patients should be transferred to LTACs, leading to variation in transfer timing by LTAC type. Currently there are no universally accepted criteria to define eligibility for LTAC transfer [3]. This opens the possibility that LTACs are selecting patients based on factors other than clinical appropriateness. Our study suggests that co-located LTACs are indeed selecting different types of patients for admission. This finding also shows that acute care hospitals with greater access to LTACs may be more profitable under prospective payment, since they are able to discharge patients earlier, spending less money but receiving the same fixed payments. Prior studies show that hospitals that host co-located LTACs tend to experience shorter lengths of stay and lower costs for ICU patients [23], supporting the notion that co-located LTACs approach patients differently at their host hospitals compared to other hospitals. Together, these results highlight the need to better standardize LTAC admission criteria, a process that is currently underway. Unfortunately, our data are not granular enough to determine the clinical appropriateness of transfer in this study.

Overall, our study implies a tradeoff between these two types of post-acute care facilities, with a small increased risk of mortality exchanged for a lower readmission rate. This tradeoff potentially suggests that the best LTAC model may be one that combines elements from both approaches, LTACs that are tightly linked to short-stay hospitals facilitating resource sharing (similar to co-located LTACs), but also large enough to provide complex, multidisciplinary care to a broad range of patients (similar to free-standing LTACs). This model is not fully supported by available evidence and should be rigorously tested if implemented. Medicare and other policy makers have the opportunity to encourage such a model as they revisit LTAC certifications and reimbursement schemes under the Affordable Care Act. Through bundled payment mechanisms Medicare could encourage LTACs to partner with short stay hospitals to avoid readmissions, while at the same time creating standardized admission criteria to prevent overutilization. The resulting larger LTACs could function as regional referral centers for chronic critical illness, providing highly skilled care for patients with chronic critical illness with increased efficiency compared to the current models.

Our study also has implications for the 25% rule, which if fully implemented would limit LTAC admissions from a single host hospital. On the one hand, given increased mortality at co-located LTACs, it might seem prudent to limit admissions to these hospitals. On the other hand, the much larger need to standardize admission criteria across LTACs is not addressed by the 25% rule, indicating a need to better define criteria for LTAC admission regardless of the type of the LTAC or the relationship between the LTAC and the short-stay hospital.

Our study has several limitations. First, we studied only Medicare beneficiaries, which are only a portion of LTAC admissions. However, these patients account for nearly 70% of LTAC use, and Medicare is the only national data source for LTAC admissions. We also performed our regression analyses on only a subset of transferred patients, a decision that increased internal validity but likely further decreased generalizability. Second, we did not have detailed clinical risk-adjustment, meaning that our findings may in part be due to unmeasured confounding. Nonetheless we used a highly homogenous cohort enhanced through propensity matching, restricted our analysis only to hospitals that transferred patients to both types of LTACs, and adjusted for many of the comorbidities known to be associated with outcomes in chronic critical illness [24]. Third, as in all observational studies we could not fully address for selection bias. Although our propensity score approach in part addressed selection by matching on likelihood to be admitted to a co-located LTAC accounting for potential differential mortality, we could not fully address selection, as might occur if co-located LTACs specifically sought certain types of patients that may have different outcomes, or the potential impact of other types of unmeasured confounding, as might occur if patients in co-located LTACs had a greater severity of illness on transfer. Fourth, we could not address confounding by the short-stay hospital of origin, as might occur if the short-stay hospitals quality is endogenous with LTAC quality. Given the separation, both fiscal and managerial, between host hospitals and co-located LTACs, and our other efforts to minimize confounding in the sample, we doubt any influence was significant. Fifth, the data for this study are now 10 years old, and may not directly related to modern LTACs utilization patterns. Unfortunately, 2005 is the last year for which reliable data on LTAC type are available. Sixth, we used total charges and cost-to-charge ratios to estimate costs. Although this approach is the only feasible way to measure costs on a national scale in the US, it may be inaccurate compared to more granular costing methodology. Our cost analysis is also limited in that MedPAR only contains inpatient costs. Although inpatient costs far exceed outpatient costs for these patients [16], we may have missed some important costs related to LTAC utilization.

## Conclusions

Our study provides new insight into the differences between free-standing and co-located LTACs. The lower risk of readmission at co-located LTACs creates uncertainty about the value of policies designed to restrict transfers to these LTACs via the 25% rule. At the same time, the higher mortality indicates the need for efforts to uncover the mechanism of the mortality difference so that we can improve the overall quality of care for patients with chronic critical illness [25].

## Supporting Information

S1 File Supporting information includes text describing the propensity matching and regression modeling, and tables that contain the demographic characteristics and outcomes of eligible patients. (DOCX)

## References

[1] Haley J, Long S. Long-term care hospitals under Medicare: facility-level characteristics. Health Care Financ Rev. 2001;23: 1–18. PMC4194716 12500335

[2] Coulam RF, Gaumer GL. Medicare's prospective payment system: a critical appraisal. Health Care Financ Rev Annu Suppl. 1991;: 45–77. 10128704

[3] Carson SS. Know your long-term care hospital. Chest. 2007;131: 2–5. 10.1378/chest.06-2513 17218547

[4] Scheinhorn DJ, Hassenpflug MS, Votto JJ, Chao DC, Epstein SK, Doig GS, et al Post-ICU mechanical ventilation at 23 long-term care hospitals: a multicenter outcomes study. Chest. 2007;131: 85–93. 10.1378/chest.06-1081 17218560

[5] Nelson JE, Cox CE, Hope AA, Carson SS. Chronic critical illness. American Journal of Respiratory and critical care medicine. 2010;182: 446–454. 10.1164/rccm.201002-0210CI 20448093 PMC2937238

[6] Pilcher DV, Bailey MJ, Treacher DF, Hamid S, Williams AJ, Davidson AC. Outcomes, cost and long term survival of patients referred to a regional weaning centre. Thorax. 2005;60: 187–192. 10.1136/thx.2004.026500 15741433 PMC1747325

[7] Schönhofer B, Euteneuer S, Nava S, Suchi S, Köhler D. Survival of mechanically ventilated patients admitted to a specialised weaning centre. Intensive Care Med. 2002;28: 908–916. 10.1007/s00134-002-1287-5 12122529

[8] Kahn JM, Benson NM, Appleby D, Carson SS, Iwashyna TJ. Long-term acute care hospital utilization after critical illness. JAMA. 2010;303: 2253–2259. 10.1001/jama.2010.761 20530778 PMC3094575

[9] Long-term care hospital services. Report to the congress: Medicare payment policy. Washington, D.C.: MedPAC; 2013.

[10] Kahn JM, Le T, Angus DC, Cox CE, Hough CL, White DB, et al The epidemiology of chronic critical illness in the United States. Crit Care Med. 2015;43: 282–287. 10.1097/CCM.0000000000000710 25377018 PMC7901538

[11] Kosecoff J, Kahn KL, Rogers WH, Reinisch EJ, Sherwood MJ, Rubenstein LV, et al Prospective payment system and impairment at discharge. The “quicker-and-sicker” story revisited. JAMA. 1990;264: 1980–1983. 2214063

[12] Kahn JM, Werner RM, Carson SS, Iwashyna TJ. Variation in long-term acute care hospital use after intensive care. Med Care Res Rev. 2012;69: 339–350. 10.1177/1077558711432889 22311957 PMC5503694

[13] Robinson JC, Casalino LP. Vertical integration and organizational networks in health care. Health Aff (Millwood). 1996;15: 7–22. 10.1377/hlthaff.15.1.7 8920566

[14] Kahn JM, Goss CH, Heagerty PJ, Kramer AA, O'Brien CR, Rubenfeld GD. Hospital volume and the outcomes of mechanical ventilation. N Engl J Med. 2006;355: 41–50. 10.1056/NEJMsa053993 16822995

[15] Kahn JM, Iwashyna TJ. Accuracy of the discharge destination field in administrative data for identifying transfer to a long-term acute care hospital. BMC Res Notes. 2010;3: 205 10.1186/1756-0500-3-205 20663175 PMC2917437

[16] Riley GF. Administrative and claims records as sources of health care cost data. Medical care. 2009;47: S51–5. 10.1097/MLR.0b013e31819c95aa 19536019

[17] Thoemmes FJ, West SG. The use of propensity scores for nonrandomized desgins with clustered data. Multivariate Behavioral Research. 2011;46: 514–543. 26735885 10.1080/00273171.2011.569395

[18] Dehejia RH, Wahba S. Propensity score-matching methods for nonexperimental causal studies. The Review of Economics and Statistics. 2002;48: 151–161.

[19] Rosenbaum PR, Rubin DB. Constructing a control group using multivariate matched sampling methods that incorporate the propensity score. The American Statistician. 1985;39: 33–38.

[20] Wilcox ME, Chong CAKY, Niven DJ, Rubenfeld GD, Rowan KM, Wunsch H, et al Do Intensivist Staffing Patterns Influence Hospital Mortality Following ICU Admission? A Systematic Review and Meta-Analyses. Crit Care Med. 2013;41: 2253–2274. 10.1097/CCM.0b013e318292313a 23921275

[21] Kim MM, Barnato AE, Angus DC, Fleisher LA, Fleisher LF, Kahn JM. The effect of multidisciplinary care teams on intensive care unit mortality. Arch Intern Med. 2010;170: 369–376. 10.1001/archinternmed.2009.521 20177041 PMC4151479

[22] Girard TD, Kress JP, Fuchs BD, Thomason JWW, Schweickert WD, Pun BT, et al Efficacy and safety of a paired sedation and ventilator weaning protocol for mechanically ventilated patients in intensive care (Awakening and Breathing Controlled trial): a randomised controlled trial. Lancet. 2008;371: 126–134. 10.1016/S0140-6736(08)60105-1 18191684

[23] Dobson A, Koenig L, Siegel J. The clinical and economic impacts of long-term hospitals. West Hartford, CT: National Association of Long Term Hospitals; 2004.

[24] Carson SS, Kahn JM, Hough CL, Seeley EJ, White DB, Douglas IS, et al A multicenter mortality prediction model for patients receiving prolonged mechanical ventilation. Crit Care Med. 2012;40: 1171–1176. 10.1097/CCM.0b013e3182387d43 22080643 PMC3395423

[25] Kahn JM, Carson SS. Generating evidence on best paractice in long-term acute care hospitals. JAMA. 2013;309: 719–720. 10.1001/jama.2013.848 23423418

