# Supplementary material for: A Comparison of Free-Standing versus Co-Located Long-Term Acute Care Hospitals
Source: PLoS One. 2015 Oct 6;10(10):e0139742. doi: 10.1371/journal.pone.0139742 (PMC4595282; doi:10.1371/journal.pone.0139742)
Supplement: S1 File — (DOCX) [file pone.0139742.s001.docx]

**A comparison of free-standing versus co-located long-term acute care hospitals**

Kahn, et. al.

SUPPORTING INFORMATION

**1. INTRODUCTION**

This supplement contains additional information for the manuscript entitled “A comparison of free-standing versus co-located long-term acute care hospitals.” The supporting information has two components:

- Supplemental methods that describe the propensity matching and regression modeling (pages 2 through 4);
- Supplemental results that contain demographic characteristics and outcomes of eligible patients (pages 5 through 8).

**2. SUPPLEMENTAL METHODS**

In this section we provide additional detail about our regression approach, including the conceptual model, the propensity score development, and the multivariable regression analysis.

**2.1 Conceptual model**. Our overall goal was to examine the relationship between LTACH hospital type (free-standing or co-located) and patient outcomes (mortality, readmission and costs). We recognized that there are two major threats to unbiased comparisons between patients admitted to free-standing and co-located LTACHs. The first threat is confounding, stemming the from measurable differences in patient characteristics across LTACH types. We addressed this threat through restriction to a homogenous cohort and multivariable modeling, which is described in the primary manuscript. The second threat is selection (i.e. endogeneity), stemming from the fact that hospitals containing co-located LTACHs are both more likely to send patients to LTACHs and more likely to send patients to co-located LTACHs. To the degree that hospital of origin is associated with outcome, LTACH type would be associated with outcomes independent of actual quality. We addressed this threat using both restriction and a propensity-score approach, which we describe in detail here.

First, we restricted the analysis only to hospitals that admitted patients to both types of LTACHs, thus substantially reducing the likelihood that hospital quality was associated with LTACH type by ensuring that no hospital only sent patients to only one kind of LTACH. Second, we developed a propensity score for transfer to a co-located LTACH among eligible patients. Importantly, we developed this score in a multivariate model with six potential outcomes: death/hospice, home, skilled nursing facility, another acute care hospital, co-located LTAC, and free-standing LTACH. Thus, the score represented the probability of transfer to a co-located LTAC accounting for the entire range of potential outcomes, not just transfer to an LTACH. Third, we matched on this propensity score only among patients actually transferred to an LTACH, resulting in a cohort who all were transferred to an LTACH but had an equal probability of transfer to a co-located LTACH. Fourth, we performed multivariable regression in our matched cohort to obtain our final results.

As for all propensity scores the goal was not to completely address selection bias and unmeasured confounding but to help make meaningful comparisons between groups by estimating propensity to be admitted to a given LTAC type. We argue that this comparison is best carried out by allowing for all possible outcomes rather than only focusing on LTAC type, as this allows for the full range of possibilities. An alternative approach would be to restrict the analysis to only patients admitted to an LTAC and then perform either propensity matching or traditional regression. However, this approach would not account for the possibility of no admission to an LTAC. By fitting a propensity model *prior* to restriction, the model accounts for differential likelihood of death or SNF transfer among some patients (since those are potential outcomes in the model).

**2.2 Propensity score development.** To create our propensity score we used all 391,292 patients in the analytic cohort (primary manuscript, Figure 1). We fit a multinomial logistic regression model where the dependent variable was hospital discharge location (categorized into six groups as defined above) and the independent variables were all patient and hospital factors potentially associated with LTACH transfer, including: age, gender, race (black, white, other), hospital admission source (emergency department, other hospital, direct); ventilated during hospital admission (yes, no); hospitalization diagnosis related group as set of indicator covariates, US census region; hospital ownership (for-profit, non-profit, government); number of LTAC beds in the Dartmouth Atlas Hospital Referral Region; and distance to the nearest LTACH in miles. We used generalized estimating equations to cluster on hospital. The pseudo-area under the Receiver Operating Characteristic curve for this model was 0.71, indicating acceptable model fit. Based on this model, we then obtained our propensity score by calculating each patient’s probability of transfer to a co-located LTACH.

We checked the validity of our model in two ways. First, we plotted histograms of our propensity score by actual transfer destination, checking to ensure that there was enough overlap for successful matching. Second, we examined patient characteristics by LTACH transfer type, stratified by decile of propensity score, checking to make sure that key patient characteristics were balanced. Together, these analyses suggested a valid propensity score.

**2.3. Multivariable regression analysis.** We limited the analysis to only patients who were actually transferred to an LTACH, and matched on the propensity score as described in the primary manuscript. We then fit a series of multivariable regression models in which the dependent variables were mortality, readmission rates and costs. We used relative risk regression for mortality, Poisson regression for readmission rates and linear regression for costs. We chose not to transform costs for this analyses, wince the central limit theorem allows for unbiased inference about adjusted means when samples sizes are large. For all the models the independent variables were age, gender, admission source, comorbidities defined in the manner of Elixhauser and modeled as a series of indicator covariates, the presence of mechanical ventilation at the acute care hospital; and whether or not the originating hospital contained a co-located LTAC, and total annual admission volume.

In these models there were two potential levels of clustering to consider: clustering at the level of the matched pair and clustering at the level of the hospital. These levels were non-nested and thus could not easily be considered simultaneously. Therefore we fit models using GEE that accounted for either no clustering, clustering by matched pair or clustering by hospital. We found that clustering by matched pair had a negligible effect on our standard errors, while clustering by hospital had a substantial effect. Thus, the final models only account for clustering by hospital.**3. SUPPLEMENTAL RESULTS**

In this section we describe complete demographic and clinical characteristics for the 391,292 eligible patients. These patients were ≥65 years of age and were admitted to an eligible short stay hospital with one of the top 10 diagnosis related groups (DRGs) resulting in an LTACH transfer. We examined demographic and clinical characteristics and outcomes for these patients categorized by discharge destination: dead/hospice, home, skilled nursing facility, other short stay hospital, free-standing LTAC, or co-located LTAC. We further categorized these patients by whether or not their originating hospital contained a co-located LTAC. The goal of this analysis was not only to understand differences in patients between the two types of LTACs, but also to examine for potential selection bias between different types of originating hospitals.

Supplemental Figure 2.1 shows the ultimate discharge location for patients with the top 10 DRG diagnoses, after exclusions, by originating hospital type. The majority of patients were either discharged home or transferred to a skilled nursing facility. Patient characteristics and outcomes are shown in Tables 2.2 (for hospitals without a co-located LTAC) and 2.3 (for hospitals with a co-located LTAC). The last two columns in each table contain similar data as Table 2 in the primary manuscript. The difference between these tables and Table 2 is that these tables show all eligible patients, not just those transferred to an LTACH. Differences in patients across discharge location (columns within each table) and across originating hospital type (columns across the two tables) might indicate sources of selection bias. Our analyses addressed this potential bias by calculating a propensity score for LTACH based on a multivariate model using all these patients.

**Supplemental Figure 2.1.** Discharge destination by originating hospital type (n=391.292). The last set of columns in the figure show the same patients as Figure 2 in the primary manuscript, except they are not broken down by LTACH type. SNF = skilled nursing facility; LTACH = long-term acute care hospital.

**
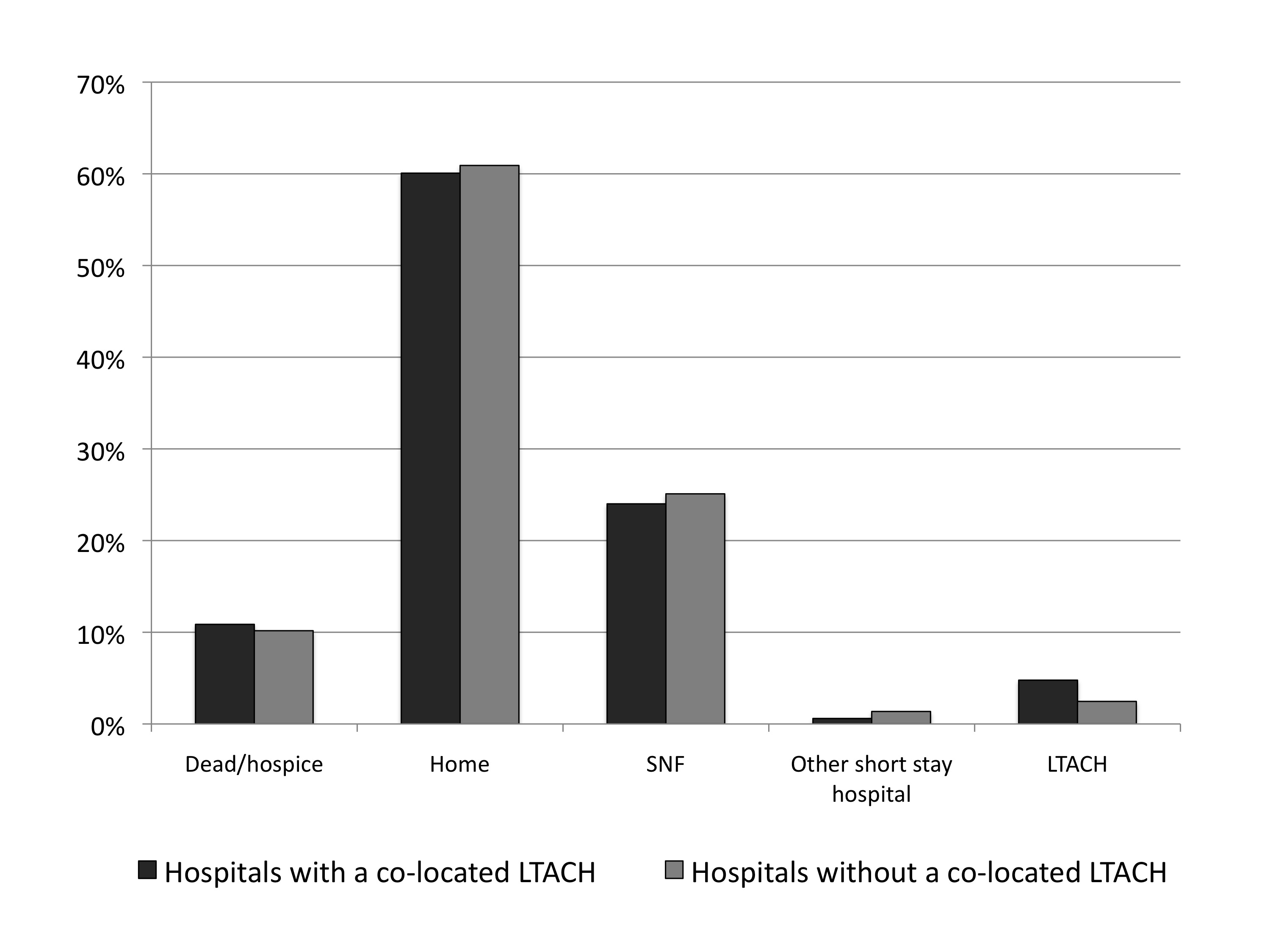
Supplemental Table 2.1.** Patient characteristics by short-stay hospital outcome for patients admitted to 650 hospitals without a co-located long-term acute care hospital

| **Variable** | **Dead/Hospice (n=32,492)** | **Home**  **(n=194,613)** | **SNF**  **(n=80,184)** | **Other short stay hospital (n=4,352)** | **Free-standing LTAC (n=5,220)** | **Co-located LTAC (n=2,638)** |
| --- | --- | --- | --- | --- | --- | --- |
| Age | 82.3 ± 8.1 | 78.2 ± 7.6 | 82.9 ± 7.8 | 77.5 ± 7.2 | 78.9 ± 7.6 | 77.6 ± 7.2 |
| Female gender | 18,367 (56.5) | 107,857 (55.4) | 51,643 (64.4) | 2,104 (48.4) | 2,977 (57.0) | 1,376 (52.2) |
| Race  White  Black  Other | 27,473 (84.6)  3,971 (12.2)  1,048 (3.2) | 160,890 (82.7)  25,939 (13.3)  7,784 (4.0) | 67,162 (83.8)  10,645 (13.3)  2,377 (3.0) | 3,630 (83.4)  548 (12.6)  174 (4.0) | 3,934 (75.4)  1,013 (19.4)  273 (5.2) | 2,134 (80.9)  387 (14.7)  117 (4.4) |
| Admission Source  Other hospital  Direct admission  ED  SNF  Other | 909 (2.8)  5,356 (16.5)  25,145 (77.4)  1,009 (3.1)  73 (0.2) | 2,052 (1.1)  55,039 (28.3)  136,522 (70.2)  695 (0.4)  305 (0.2) | 1,245 (1.6)  13,479 (16.8)  61,442 (76.6)  3,874 (4.8)  144 (0.2) | 79 (1.8)  949 (21.8)  3,279 (75.3)  39 (0.9)  6 (0.1) | 205 (3.9)  982 (18.8)  3,947 (75.6)  71 (1.4)  15 (0.3) | 133 (5.0)  665 (25.2)  1,790 (67.9)  42 (1.6)  8 (0.3) |
| ICU admission | 16,784 (51.7) | 23,945 (12.3) | 16,852 (21.0) | 1,725 (39.6) | 3,043 (58.3) | 1,876 (71.1) |
| Mechanical ventilation | 12,132 (37.3) | 4,924 (2.5) | 6,231 (7.8) | 757 (17.4) | 2,368 (45.4) | 1,691 (64.1) |
| Length of stay  ICU  Hospital | 5.4 ± 10.5  9.2 ± 13.1 | 1.5 ± 3.1  5.3 ± 4.8 | 3.1 ± 6.9  8.9 ± 9.0 | 3.7 ± 8.4  6.7 ± 11.2 | 12.9 ± 14.5  17.8 ± 14.5 | 18.6 ± 17.1  23.4 ± 17.0 |
| Final Destination  Home  Acute Care  SNF  Dead  Hospice | n/a | n/a | n/a | n/a | 1,598 (30.6)  650 (12.5)  1,885 (36.1)  957 (18.3)  130 (2.5) | 594 (22.5)  275 (10.4)  1,134 (43.0)  569 (21.6)  66 (2.5) |

SNF = skilled nursing facility; LTAC = long-term acute care hospital; ED = emergency department; ICU = intensive care unit

**Supplemental Table 2.2.** Patient characteristics by short-stay hospital outcome for patients admitted to 98 hospitals with a co-located long-term acute care hospital

| **Variable** | **Dead/Hospice (n=7,337)** | **Home**  **(n=40,571)** | **SNF**  **(n=16,213)** | **Other short stay hospital (n=404)** | **Free-standing LTAC (n=589)** | **Co-located LTAC* (n=2,421)** |
| --- | --- | --- | --- | --- | --- | --- |
| Age | 82.0 ± 8.2 | 78.0 ± 7.5 | 82.7 ± 7.8 | 77.3 ± 7.1 | 78.5 ± 7.6 | 78.7 ± 7.5 |
| Female gender | 4,192 (57.1) | 22,473 (55.4) | 10,570 (65.2) | 183 (45.3) | 324 (55.0) | 1,316 (54.4) |
| Race  White  Black  Other | 5,977 (81.5)  1,079 (14.7)  281 (3.8) | 32,509 (80.1)  6,370 (15.7)  1,692 (4.1) | 13,123 (80.9)  2,531 (15.6)  559 (3.5) | 319 (79.0)  76 (18.8)  9 (2.2) | 456 (77.4)  98 (16.6)  35 (6.0) | 1,790 (73.9)  544 (22.5)  87 (3.6) |
| Admission Source  Other hospital  Direct admission  ED  SNF  Other | 277 (3.8)  992 (13.5)  5,819 (79.3)  246 (3.4)  3 (0.0) | 544 (1.3)  10,103 (24.9)  29,736 (73.3)  183 (0.5)  5 (0.0) | 364 (2.3)  2,145 (13.2)  12,817 (79.1)  884 (5.5)  3 (0.0) | 14 (3.5)  62 (15.4)  325 (80.5)  3 (0.7)  0 (0.0) | 27 (4.6)  116 (19.7)  432 (73.3)  14 (2.4)  0 (0.0) | 105 (4.3)  561 (23.2)  1,720 (71.1)  35 (1.5)  0 (0.0) |
| ICU admission | 3,910 (53.2) | 5,482 (13.5) | 3,512 (21.7) | 193 (47.8) | 343 (58.2) | 1,579 (65.2) |
| Mechanical ventilation | 2,706 (36.9) | 1,098 (2.7) | 1,054 (6.5) | 91 (22.5) | 238 (40.4) | 1,149 (47.5) |
| Length of stay  ICU  Hospital | 5.0 ± 9.7  8.6 ± 12.2 | 1.5 ± 3.0  5.4 ± 4.3 | 2.7 ± 5.6  8.5 ± 7.7 | 3.7 ± 6.2  7.1 ± 8.7 | 11.1 ± 13.8  16.5 ± 14.7 | 11.5 ± 12.3  17.0 ± 12.4 |
| Final Destination  Home  Acute Care  SNF/Rehab  Dead  Hospice | n/a | n/a | n/a | n/a | 205 (34.8)  76 (12.9)  198 (33.6)  93 (15.8)  17 (2.9) | 692 (28.6)  214 (8.8)  894 (36.9)  541 (22.4)  80 (3.3) |

* Patients admitted to the LTAC co-located within the admitting short stay hospital. Excludes 216 patients admitted to an LTAC that is co-located within a different short-stay hospital.

SNF = skilled nursing facility; LTAC = long-term acute care hospital; ED = emergency department; ICU = intensive care unit

**Supplemental Table 2.3**. Patient characteristics for the cohort of patients transferred to long-term acute care hospitals and eligible for the propensity match.

|  | **Eligible Sample** | |
| --- | --- | --- |
|  | **Free-standing**  **(n=5,809)** | **Co-located**  **(n=5,059)** |
| Age | 78.9 ± 7.6 | 78.5 ± 7.5 |
| Female (%) | 3,301 (56.8) | 2,829 (55.9) |
| Race  White  Black  Other | 4,390 (75.6)  1,111 (19.1)  308 (5.3) | 3,818 (75.5)  969 (19.2)  272 (5.3) |
| Admitted to ICU at short stay hospital | 3,586 (61.7) | 3,262 (64.5) |
| Mechanical ventilation status  Short stay only  Short stay and LTAC  Neither | 1,071 (18.4)  1,612 (27.8)  3,126 (53.8) | 966 (19.1)  1,874 (37.0)  2,170 (42.9) |
| Length of stay in short stay hospital  ICU  Hospital | 13.3 ± 14.1  17.6 ± 14.5 | 14.1 ± 14.9  18.9 ± 15.0 |

ICU = intensive care unit; LTAC = long-term acute care hospital
